# Treatment modalities in recurrent brain metastases: a combined institutional and individual patient data meta-analysis of post-recurrence survival and local progression-free survival

**DOI:** 10.1007/s11060-025-05244-1

**Published:** 2025-11-25

**Authors:** Alim Emre Basaran, Luca Fahsold, Florian Lordick, Nils H. Nicolay, Erdem Güresir, Johannes Wach

**Affiliations:** 1 Department of Neurosurgery, University Hospital Leipzig, Leipzig University, Liebigstr. 20, 04103 Leipzig, Germany; 2 Comprehensive Cancer Center Central Germany, Partner Site Leipzig, 04103 Leipzig, Germany; 3 Department of Medicine (Oncology, Gastroenterology, Hepatology, Pulmonology), University of Leipzig Medical Center, Cancer Center Central Germany, 04103 Leipzig, Germany; 4 Department of Radiation Oncology, University Leipzig Medical Center, Stephanstraße 9a, 04103 Leipzig, Germany

**Keywords:** Recurrent brain metastases, Salvage re-resection, Adjuvant radiotherapy, Stereotactic radiosurgery, Post-recurrence survival, Progression free survival

## Abstract

**Background:**

Brain metastases (BM) are among the most common intracranial tumors. Despite advances in multimodal therapy for newly diagnosed BM, the management of recurrent BM remains a clinical challenge. Due to the lack of robust data, there is currently no consensus regarding optimal salvage treatment for recurrent BM.

**Methods:**

Institutional data (2016–2025) and published data from the literature (2011–2025) were analyzed with respect to overall survival (OS) and progression-free survival (PFS) after recurrence. Survival data were extracted from Kaplan-Meier curves of the selected studies using the R package IPDfromKM and pooled survival analyses were performed.

**Results:**

In a pooled analysis of 776 patients, local surgical re-resection after recurrence was associated with significantly longer survival compared to both non-surgical management (median 14.74 [95% CI: 11.68–17.80] vs. 10.34 months [95% CI: 8.59–12.08]; HR: 0.664; *p* < 0.001) and only repeat stereotactic radiosurgery (Re-SRS) (median 14.74 months [95% CI: 10.51–18.98] vs. 10.97 months [95% CI: 9.1–12.84]; HR: 0.62; *p* < 0.001). Among patients who underwent local re-resection, gross total resection (GTR) led to markedly improved OS compared to subtotal or incomplete resection (median 23.97 months [95% CI: 15.95–31.99] vs. 7.06 months [95% CI: 5.21–8.90]; HR: 0.400; *p* < 0.0001). The addition of adjuvant re-radiotherapy after re-resection did not result in a significant survival benefit (*p* = 0.357). Regarding PFS, patients treated with local re-resection alone had the longest median PFS (43.23 months), significantly outperforming both those receiving re-resection plus adjuvant re-SRS (29.92 months; HR = 0.529; *p* < 0.001) and those treated with Re-SRS alone (15.79 months; HR = 3.031; *p* < 0.001).

**Conclusions:**

This study highlights the role of local re-resection in improving survival among patients with recurrent brain metastases amenable to repeat GTR. Re-SRS remains a valuable salvage option, particularly for patients in whom GTR is not feasible. While adjuvant re-radiotherapy following re-resection did not demonstrate a clear survival advantage in our analysis, it may offer additional local control in selected cases. These findings emphasize the importance of individualized, multidisciplinary decision-making to tailor salvage strategies to patient- and tumor-specific factors.

## Graphical Abstract

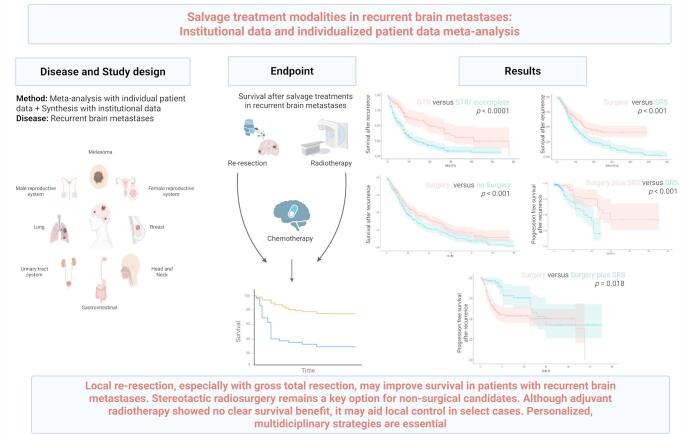

## Introduction

Brain metastases (BM) are among the most common malignant tumors of the central nervous system. Approximately 20% of all cancer patients develop BM [1, 2]. Advances in medical treatment and novel therapeutic options have improved survival and local control [3–6]. However, as overall survival (OS) increases, the risk of recurrent BM rises, posing a therapeutic challenge [7, 8].

While initial treatment of BM is well established and typically involves a multimodal approach including surgery, adjuvant radiotherapy (stereotactic radiosurgery [SRS] or whole-brain radiotherapy [WBRT]), and systemic therapy, the management of recurrent BM remains unclear. Therapeutic decision making is individualized and depends on factors such as the Karnofsky Performance Score (KPS), tumor size, neuroanatomical location, primary tumor type and prior treatments. In addition, prior therapies, radiation dose and fractionation, and the interval to recurrence can influence both efficacy and toxicity of salvage options [9–11]. To date, no randomized controlled trials have directly compared repeat surgical resection with or without adjuvant radiotherapy.

The aim of the present study was to systematically evaluate treatment strategies for recurrent BM including surgical re-resection, re-SRS, and adjuvant systemic therapies with respect to their impact on OS and progression-free survival (PFS) after recurrence. To this end, we analyzed institutional data and a meta-analysis of longitudinal data using reconstructed individual patient data (IPD) from published Kaplan-Meier curves. We also examined whether the extent of resection (gross-total vs. subtotal/ incomplete) is associated with survival outcomes. By combining an institutional cohort with IPD reconstructed from the literature, this study seeks to provide comparative effectiveness data to inform multidisciplinary decision making in the recurrent BM setting.

## Materials and methods

### Search strategy and data collection

Data from included studies were extracted from Pubmed, Google Scholar and Cochrane Library from January 1, 2011, to June 1, 2025. The following MeSH terms and strategy were used: brain neoplasms, brain metastases, brain metastasis, and neoplasm recurrence. Search strategy: (“Brain Neoplasms/secondary“[MeSH] OR “brain metastases“[tiab] OR “cerebral metastases“[tiab]) AND (“recurrence“[tiab] OR “recurrent“[tiab] OR “Local“[MeSH]) AND (“radiosurgery“[tiab] OR “stereotactic radiosurgery“[tiab] OR “radiotherapy“[tiab] OR “re-irradiation“[tiab] OR “re-radiotherapy“[tiab] OR “repeat radiotherapy“[tiab] OR “repeat radiosurgery“[tiab] OR “re-stereotactic radiosurgery“[tiab]) AND (“Treatment Outcome“[MeSH] OR “outcome“[tiab] OR “efficacy“[tiab] OR “survival“[tiab] OR “local control“[tiab]).

### Data extraction and individual patient data reconstruction

Two independent authors (AEB, JW) extracted the following data from the included studies: year of publication, number of patients, extent of resection (EoR) (if available), median age of patients, sex, neuroanatomical location (if available), initial therapy at first diagnosis (if available), primary tumor origin (if available), treatment modality at recurrence, adjuvant radiotherapy following local re-resection, PFS and OS after recurrence.

For the studies by Holt et al. [12], Cummins et al. [13], Buszek et al. [14], Lin et al. [15], Maranzano et al. [16], Rava et al. [17], Touati et al. [18], Wilcox et al. [19], Wasilewski et al. [20], McKay et al. [21], Noguchi et al. [22] and Yomo et al. [23] IPD were reconstructed from Kaplan-Meier curves and the corresponding number-at-risk tables. Curves were extracted using Digitizelt software (version 2.5.10, macOS), and IPD reconstruction was performed using the IPDfromKM R package [24, 25]. 

### Inclusion criteria for included studies

Studies were eligible if they enrolled adults (≥ 18 years) with recurrent parenchymal brain metastases confirmed by neuroimaging and/or histopathology. Eligible English-language reports provided post-recurrence OS and/or PFS with extractable Kaplan–Meier curves and corresponding numbers-at-risk, and specified the salvage modality (re-resection, re-SRS, or re-resection plus adjuvant SRS). We excluded leptomeningeal-only relapse, case reports or small series without time-to-event outcomes, and studies that did not distinguish post-recurrence outcomes. For overlapping publications from the same center, we retained the most comprehensive nonredundant dataset. Studies reporting only re-SRS after recurrence were eligible for comparison with re-resection if outcomes were reported separately by modality. Series that pooled modalities without separable outcomes were excluded.

#### Study design and ethics

We conducted a two-component study comprising a retrospective institutional cohort of patients treated for recurrent BM and a systematic review with reconstruction of IPD from published Kaplan-Meier curves. The requirement for written informed consent was waived by the Ethics Committee of the Medical Faculty of the University of Leipzig (approval number 296/25-ek) due to the retrospective nature of the study. The systematic review adhered to the PRISMA guidelines and was prospectively registered with PROSPERO (ID: CRD420251055004).

### Institutional patient selection

The institutional cohort included consecutive adults (≥ 18 years) with histopathologically confirmed local recurrence of parenchymal brain metastases who were discussed at the weekly multidisciplinary board and underwent salvage re-resection and/or re-SRS at our center between January 1, 2016, and April 30, 2025. Cases were identified from electronic medical records and board logs. Inclusion required local therapy, histopathologic confirmation, documentation of the EoR for surgical cases, and follow-up sufficient to ascertain post-recurrence OS and/or PFS. Exclusions were leptomeningeal-only relapse, diffuse or multifocal disease precluding re-resection, KPS/ comorbidity precluding anesthesia or recovery, uncontrolled extracranial disease with short expected survival, lesions not amenable to safe re-resection, or inadequate follow-up. All cases were board reviewed, and the final modality was chosen by shared decision making after counseling on risks, expected symptom relief, prior radiation exposure, and patient preferences. For the detailed institutional patient-selection workflow, see Fig. 2.

### Statistical analysis

IPD extracted from Kaplan-Meier curves for PFS and OS were pooled. Statistical differences between groups were assessed using log-rank tests. Hazard ratios (HR) and 95% confidence intervals (CI) were estimated using Cox proportional hazards regression models. Statistical analyses were performed using SPSS version 29.0 (IBM, Armonk, NY, USA). Kaplan-Meier curves were generated using R software (version 4.3.1) with the *Survival* and *Survminer* packages. Statistical significance was defined as *p* < 0.05. We prespecified subgroups (lesion location, lesion size, primary diagnosis, and systemic status), but these variables were inconsistently reported or not linkable to post-recurrence survival by salvage modality. Subgroup-specific Kaplan–Meier curves or HR were rarely available and definitions were not harmonized, so strata were not pooled. Primary origin (lung, breast, melanoma, renal, gastrointestinal, other) was extracted where reported. Because origin could not be linked to survival curves, we report only descriptive origin composition by salvage modality (Tables 1 and 2).

## Results

### Search results and included studies

**Fig. 1:**
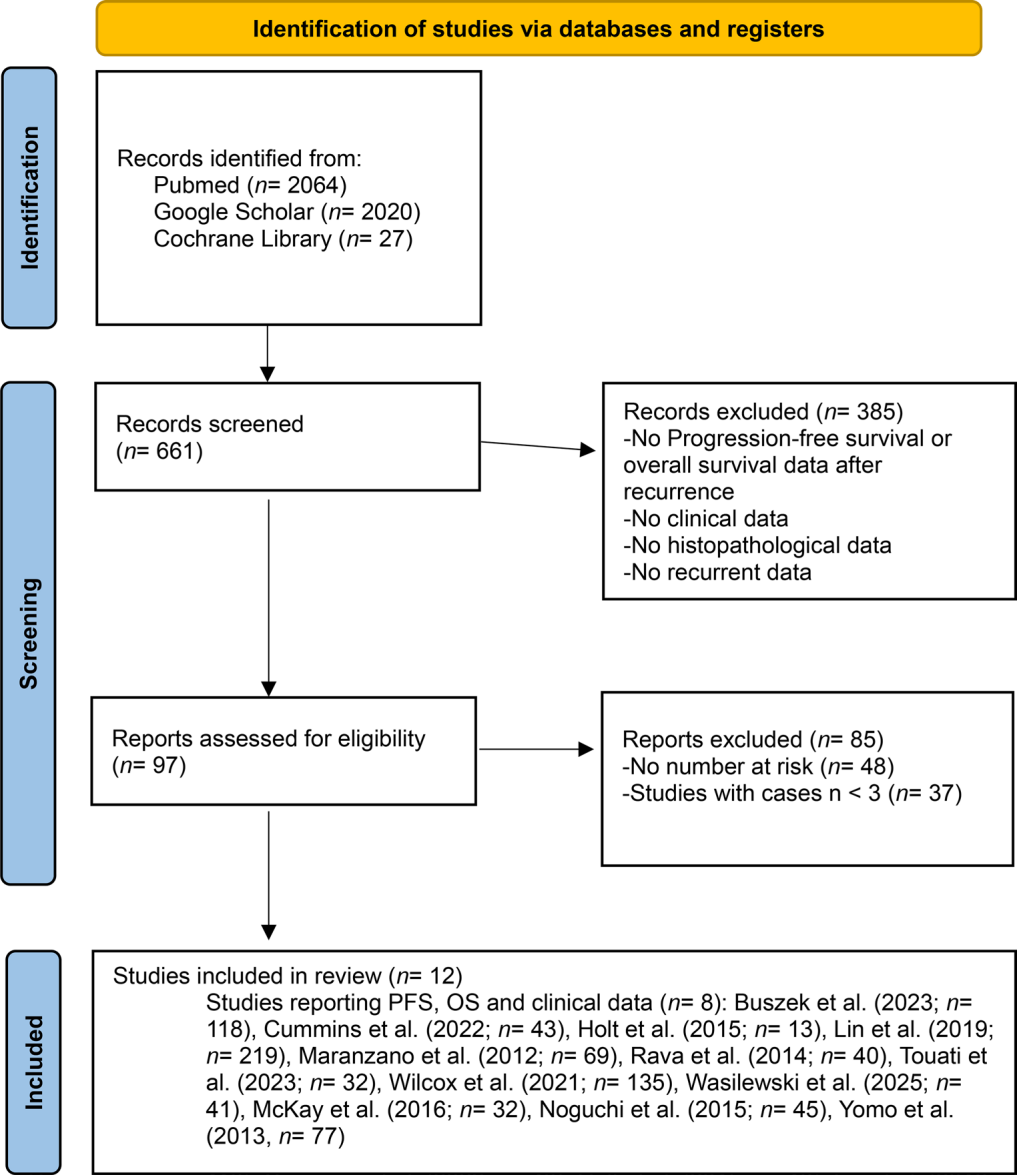
Prisma flowchart study selection

The initial systematic search of PubMed, Google Scholar, and the Cochrane Library identified 4111 studies. After title and abstract screening, 97 full-text articles were assessed for eligibility. Full texts were excluded for reasons including missing data on post-recurrence PFS or OS, insufficient clinical information, lack of confirmed pathological diagnosis, or missing documentation of salvage at recurrence. Twelve studies were included in the final meta-analysis. Studies were published between 2012 and 2025. All studies reported on the treatment approach at recurrence and subsequent adjuvant radiotherapy or observation when available. Detailed workflow and study characteristics are summarized in Fig. 1; Table 1. Subgroup synthesis by lesion location, lesion size, primary diagnosis, and systemic disease status was not feasible. In most reports these factors were incompletely documented or not separable by salvage treatment modality relative to the post-recurrence survival curves.

**Table 1:** Patient characteristics of the included studies

Study	Year	*n*	Sex (M: F)	Age Mean (range)	Neuroanatomical location	Primary tumor	Primary treatment at diagnosis	Treatment of recurrent BM	Adjuvant Therapy/ Concurrent therapy
Cummins et al.	2022	43	20:23	58.1 (33.4–84.6)	Frontal 19/43 (38%) Parietal 9/43 (18%) Occipital 10/43 (20%) Cerebellum 7/43 (14%) Temporal 5/43 (10%)	Melanoma 18/43 (36%) NSCLC 15/43 (30%) Breast 9/43 (18%) Gastrointestinal 6/43 (12%) Renal cell carcinoma 2/43 (4%)	SRS 43/43 (100%)	Re-resection 43/43 (100%)	None 21/50 (42%) Brachytherapy 16/50 (32%) SRS 23/50 (46%) Systemic therapy 23/50 (46%)
Lin et al.	2019	219	121/98	60.0 (52–69)	NA	NA	NA	No surgery 124/219 (56.6%) Re-resection 95/219 (43.4%)	None 45/219 (20.5%) SRS 74/219 (33.8%) systemic therapy 20/219 (9.2%) combined 80/219 (36.5%)
Wilcox et al.	2021	135	49/86	58 (20–92)	Frontal 48/155 (31%) Parietal 35/155 (22.6%) Occipital 20/155 (12.9%) Temporal 23/155 (14.8%) Cerebellar 29/155 (18.7%)	NSCLC 50/135 (37%) Melanoma 35/135 (25.9%) Breast 28/135 (20.7%) RCC 6/135 (4.4%) CRC 3/135 (2.2%) Other 13/135 (9.6%)	SRS 118/155 (76.1%) SRS plus Whole Brain Radiotherapy (WBRT) 12/155 (7.7%) Resection plus SRS 20/155 (12.9%) Resection plus SRS plus WBRT 5/155 (3.2%)	Re-resection 135/135 (100%)	SRS 39/155 (25.2%) Observation 116/155 (74.8%)
Buszek et al.	2023	225	119/106	52.5 (15.6–78.8)	NA	Breast 34/225 (15%) Cervical 2/225 (0.9%) Colorectal 9/225 (4%) Endometrial 2/225 (0.9%) Esophagus/GE junction 7/225 (3.1%) Head and neck 6/225 (2.7%) Melanoma 88/225 (39.1%) NSCLC 43/225 (19.1%) Prostate 2/225 (0.9%) RCC 9/225 (4%) Rectal 2/225 (0.9%) Sarcoma 12/225 (5.3%) Skin 1/225 (0.4%) SCLC 3/225 (1.3%) Testicular 1/225 (0.4%) Thyroid 1/225 (0.4%) Unknown primary 2/225 (0.9%) Urothelial 1/225 (0.4%)	SRS 225/225 (100%)	Re-resection 225/225 (100%)	SRS 21/225 (9.3%) Observation 204/225 (90.7%)
Holt et al.	2015	13	5/8	53 (30–70)	NA	Melanoma 9/13 (60%) Breast 2/13 (13.3%) Lung 1/13 (6.7%) Renal 1/13 (6.7%) Colon 1/13 (6.7%) Endometrial 1/13 (6.7%)	SRS 13/13 (100%)	Re-resection 13/13 (100%)	SRS 13/13 (100%)
Maranzano et al.	2011	69	32/37	55 (35–76)	Cerebellum 40/150 (26.7%) Frontal 34/150 (22.6%) Parietal 26/150 (17.3%) Temporal 19/150 (12.7%) Occipital 12/150 (8.0%) Brain stem 5/150 (3.3%) Thalamus 4/150 (2.7%) Other 10/150 (6.7%)	NSCLC 31/69 (45%) Breast 20/69 (29%) SCLC 9/69 (13.0%) Colorectal 7/69 (10%) Ovarian 1/69 (1.5%) Melanoma 1/69 (1.5%)	WBRT 69/69 (100%)	SRS 69/69 (100%)	Observation 48/69 (69.6%) SRS 15/69 (21.7%) Resection 2/69 (2.9%) RTX and Chemotherapy 4/69 (5.8%)
Rava et al.	2014	40	15/25	61 (36–79)	NA	NA	WBRT 27/40 (67.5%) Resection plus SRS 1/40 (2.5%) Prophylactic cranial radiation 10/40 (26%) NA 2/40 (5%)	SRS 40/40 (100%)	NA
Touati et al.	2023	32	17/15	63 (42–79)	NA	Breast 6/32 (19%) Lung 18/32 (56.3%) Melanoma 5/32 (15.6%) Prostate 1/32 (3.1%) GI 1/32 (3.1%) Unknown 1/32 (3.1%)	Resection 6/34 (18%) SRS 28/34 (82%)	SRS 32/32 (100%)	No systemic therapy 25/34 (74,0%) Chemotherapy 4/34 (11,0%) Hormonotherapy 1/34 (3,0%) Immunotherapy 2/34 (6,0%) Anti-HER-2 2/34 (6,0%) Other 13/32 (41,0%) no other SRS 19/32 (59,0%)
Wasilewski et al.	2025	41	NA	59.5 (46.3–67.1)	NA	NA	NA	Re-resection 41/41 (100%)	NA
McKay et al.	2016	32	NA	59 (36–88)	NA	NSCLC 16/32 (50%) Breast 9/32 (28%) Melanoma 2/32 (6%) RCC 2/32 (6%) Esthesioneuroblastoma 1/32 (3%) Colorectal carcinoma 1/32 (3%) SCLC 1/32 (3%)	SRS 32/32 (100%)	SRS 32/32 (100%)	NA
Noguchi et al.	2025	45	NA	NA	NA	NSCLC 22/45 (48.9%) SCLC 4/45 (8.9%) Breast 4/45 (8.9%) GI 12/ 45 (26.7%) Other 3 (6.7%)	SRS 45/45 (100%)	SRS 45/45 (100%)	No concurrent therapy 19/45 (42.2%) Concurrent therapy 26/45 (57.8%) Chemotherapy 4/26 (15.4%) Targeted therapy 16/26 (61.5%) Immuntherapy 6/26 (23.1.%)
Yomo et al.	2013	77	38/39	62 (33–82)	NA	Lung 47/77 (41%) Breast 20/77 (26%) GI 3/77 (3.9%) Others 7/77 (9.1%)	WBRT 77/77 (100%)	SRS 77/77 (100%)	NA

### Overall survival outcomes after local recurrence of brain metastases: institutional cohort analysis

**Fig. 2:**
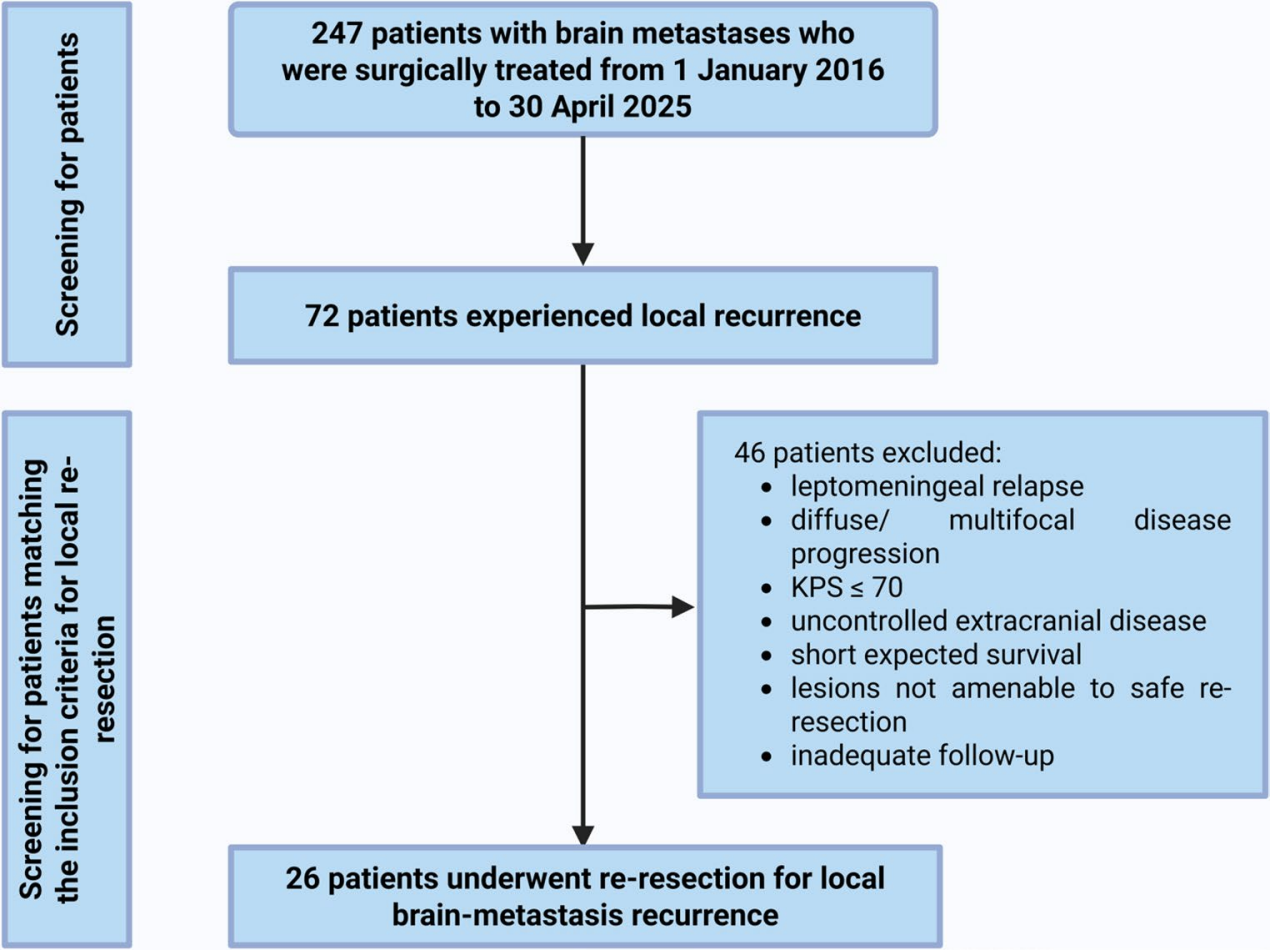
Institutional patient-selection flow diagram for salvage re-resection. From 247 patients with BM surgically treated between 1 January 2016 and 30 April 2025, 72 experienced local recurrence. Forty-six were excluded for the following reasons: leptomeningeal-only relapse, diffuse/ multifocal intracranial progression precluding local re-resection, KPS ≤70, uncontrolled extracranial disease/ short expected survival, lesions not amenable to safe re-resection, or inadequate follow-up. The final cohort comprised 26 patients who underwent re-resection for local BM recurrence

We included 26 patients from the institutional dataset were included in the analysis. Of these, 8 (30.8%) were female and 18 (69.2%) were male. At initial diagnosis, 2 patients (7.7%) received fractionated stereotactic radiotherapy (FSRT) with a total dose of 36 Gy after primary resection, while 24 patients (92.3%) underwent surgery prior to SRS. The median age at recurrence was 66 (IQR: 60–72) years. Among the 26 included patients, the primary tumor was located in the lung in 13 (50.0%), breast in 6 (23.1%), gastrointestinal tract in 3 (11.5%), and skin in 4 (15.4%). Postoperative wound healing complications occurred in 3 (11.5%), and 13 (50.0%) received postoperative adjuvant SRS. Of these, 2 (15.4%) received single-fraction SRS with 18 Gy, while 11 (84.6%) received FSRT with a total dose of 36 Gy. Regarding the Graded Prognostic Assessment (GPA), 1 (3.8%) had a GPA score of 1, 4 (15.4%) a score of 1.5, 6 (23.1%) a score of 2, 5 (19.2%) 2.5, 9 (34.6%) a score of 3 and 1 (3.8%) had a GPA score of 4. The neuroanatomical location of the recurrent brain metastases was frontal in 7 (26.9%), temporal in 5 (19.2%), parietal in 3 (11.6%), occipital in 7 (26.9%), and cerebellar in 4 (15.4%). Gross total resection (GTR) was achieved in 18 (69.2%), while 8 (30.8%) had a STR. GTR was defined as the absence of contrast-enhancing (CE) tumor on postoperative T1-weighted MRI sequences. STR was defined as the presence of residual CE tumor. Additionally, operative reports were reviewed by the operating neurosurgeon, and the classification into GTR or STR also considered intraoperative microscopic assessment of tumor remnants. See Table 2 for additional patient characteristics and Fig. 2 for the detailed workflow.

**Table 2:** Patient characteristics of our institutional data

Patient characteristics	
**Sex**	
Male	18/26 (69.2%)
Female	8/26 (30.8%)
**Age at diagnosis** (median, IQR)	66 (60–72)
**Primary tumor**	
Lung	13/26 (50.0%)
Breast	6/26 (23.0%)
Melanoma	4/26 (15.7%)
Gastrointestinal	3/26 (11.3%)
**Neuroanatomical location**	
Frontal	7/26 (26.9%)
Temporal	5/26 (19.2%)
Parietal	3/26 (11.6%)
Occipital	7/26 (26.9%)
Cerebellar	4/26 (15.4%)
**Extracranial progress**	
Yes	17/26 (65.4%)
No	9/26 (34.6%)
**Extent of resection**	
GTR	18/26 (69.2%)
STR	8/26 (30.8%)
GPA score at local recurrence	
1	1/26 (3.8%)
1.5	4/26 (15.4%)
2	6/26 (23.1%)
2.5	5/26 (19.2%)
3	9/26 (34.6%)
4	1/26 (3.8%)
**Adjuvant treatment after recurrence**	
None	13/26 (50.0%)
Radiation	8/26 (30.9%)
Radiation + Chemotherapy	3/26 (11.5%)
Radiation + Immunotherapy	1/26 (3.8%)
Immunotherapy	1/26 (3.8%)
**Postoperative complications**	
Wound healing complications	3/26 (11.5%)

### Overall survival outcomes after local recurrence in brain metastases: a pooled analysis of institutional data and IPD

For the analysis of OS after local recurrence, IPD were available for 776 patients, including 26 patients from our institutional cohort. Of the total cohort, 366 (47.2%) underwent local re-resection, while 410 (52.8%) did not receive surgical treatment. Among re-resected patients, 113 (61.1%) received no radiotherapy, while 72 (38.9%) received postoperative radiotherapy. The median OS of patients who underwent re-resection after local recurrence was 14.74 months (95% CI: 11.68–17.80), compared to 10.34 months (95% CI: 8.59–12.08) in the non-surgical group. This difference was significant (HR: 0.664; 95% CI: 0.56–0.79; *p* < 0.001). When comparing re-resection to re-SRS as salvage therapy after recurrence, re-resection resulted in a median OS of 14.74 months (95% CI. 10.51–18.98), versus 10.97 months (95% CI: 9.1-12.84) with repeat SRS (HR: 0.62, 95% CI: 0.47–0.82, *p* = 0.001).

Among patients who underwent local re-resection, those who received additional adjuvant radiotherapy had a median OS of 19.94 months (95% CI: 11.24–28.64), compared to 14.03 months (95% CI: 9.85–18.21) in those without adjuvant radiotherapy. However, this difference was not statistically significant (HR: 0.838; 95% CI: 0.58–1.22; *p* = 0.357).

**Fig. 3:**
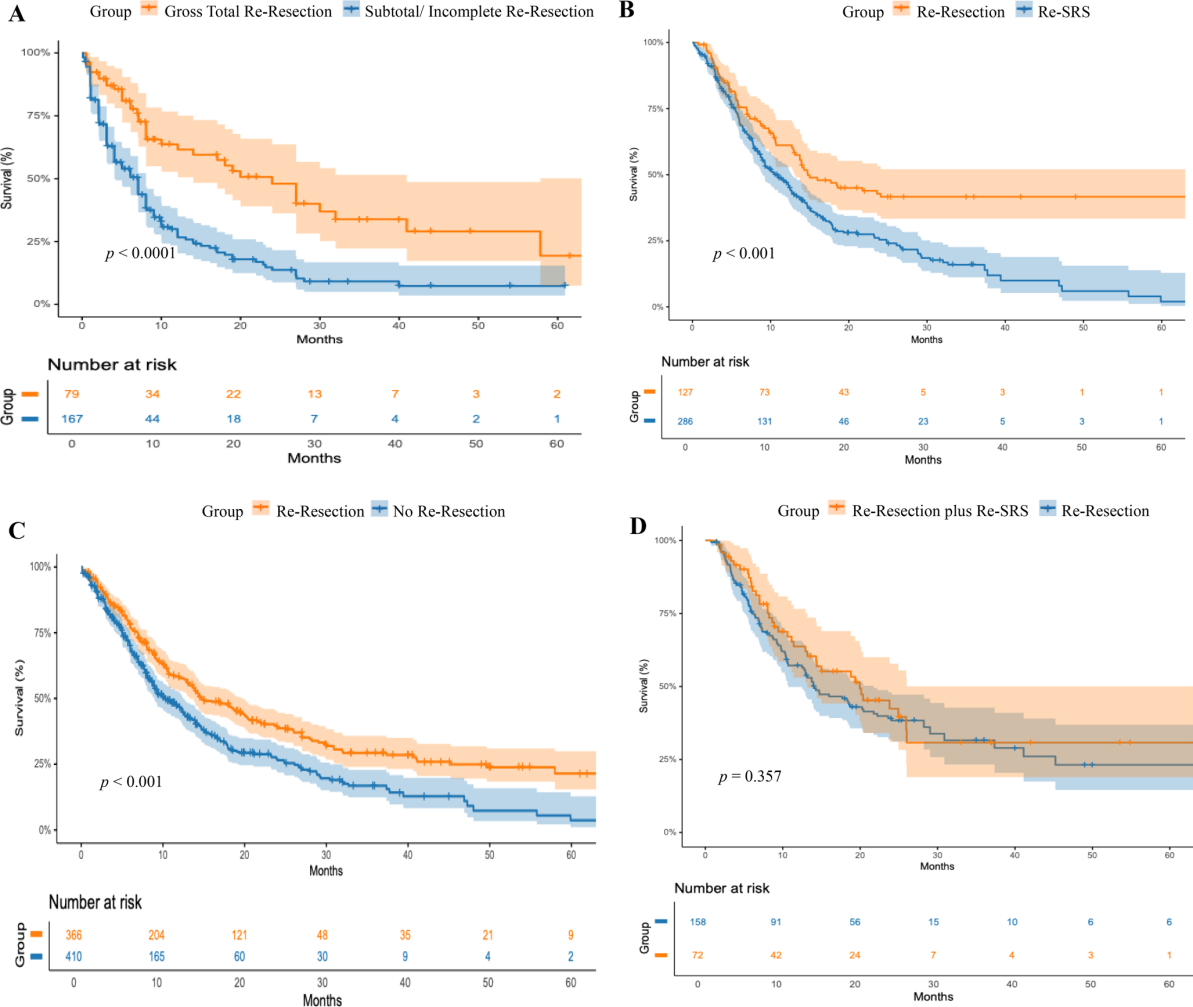
Kaplan-Meier analyses of overall survival in patients with recurrent brain metastases according to treatment modality. (**A**) Kaplan-Meier curves comparing patients who underwent local GTR re-resection after recurrence versus those with incomplete or subtotal local re-resection. Patients with GTR re-resection had a significantly longer median OS compared to those with incomplete or subtotal re-resection (23.9 vs. 7.1 months; *p* < 0.0001) after recurrence. (**B**) Kaplan-Meier curves for comparing patients treated with only local re-resection versus with only SRS. Only local re-resection was associated with significantly improved median OS compared to only SRS (14.74 vs. 10.97 months; *p* < 0.001). (**C**) Kaplan-Meier curves comparing patients who underwent local re-resection versus those who did not. The re-resection group had significantly longer OS (14.74 vs. 10.34 months; *p* < 0.001). (**D**) Kaplan-Meier curves comparing patients who received only local re-resection versus those who received local re-resection plus adjuvant SRS. Overall survival was 19.95 months in the local re-resection plus adjuvant SRS group versus 14.03 months in only re-resection group. This difference was not statistically significant (*p* = 0.357). Shaded areas indicate 95% confidence intervals (CIs). The number of patients at risk at specified time points is shown below each plot. Tumor origin is not shown on the curves because origin-specific plots were unavailable for most cohorts

With regard to the EoR, GTR was associated with a median OS of 23.97 months (95% CI: 15.95–31.99), compared to 7.06 months (95% CI: 5.21–8.90) in patients with incomplete resection. This difference was statistically significant (HR = 0.400; 95% CI: 0.278–0.580; *p* < 0.0001. Kaplan-Meier curves for OS after recurrence are shown in Fig. 3.

We compared OS across adjuvant strategies. Median OS was 10.6 months (95% CI: 7.6–13.6) in patients who received no adjuvant radiotherapy, 10.6 months (95% CI: 6.9–14.3) with radiotherapy alone, 14.0 months (95% CI: 2.2–25.7) with systemic therapy alone, and 7.8 months (95% CI: 6.5–9.1) with combined radiotherapy and systemic therapy. No significant OS differences were observed between groups (log-rank test, *p* = 0.358), and Cox regression analysis showed no significant association (HR = 1.095; 95% CI: 0.985–1.218; *p* = 0.094).

**Fig. 4:**
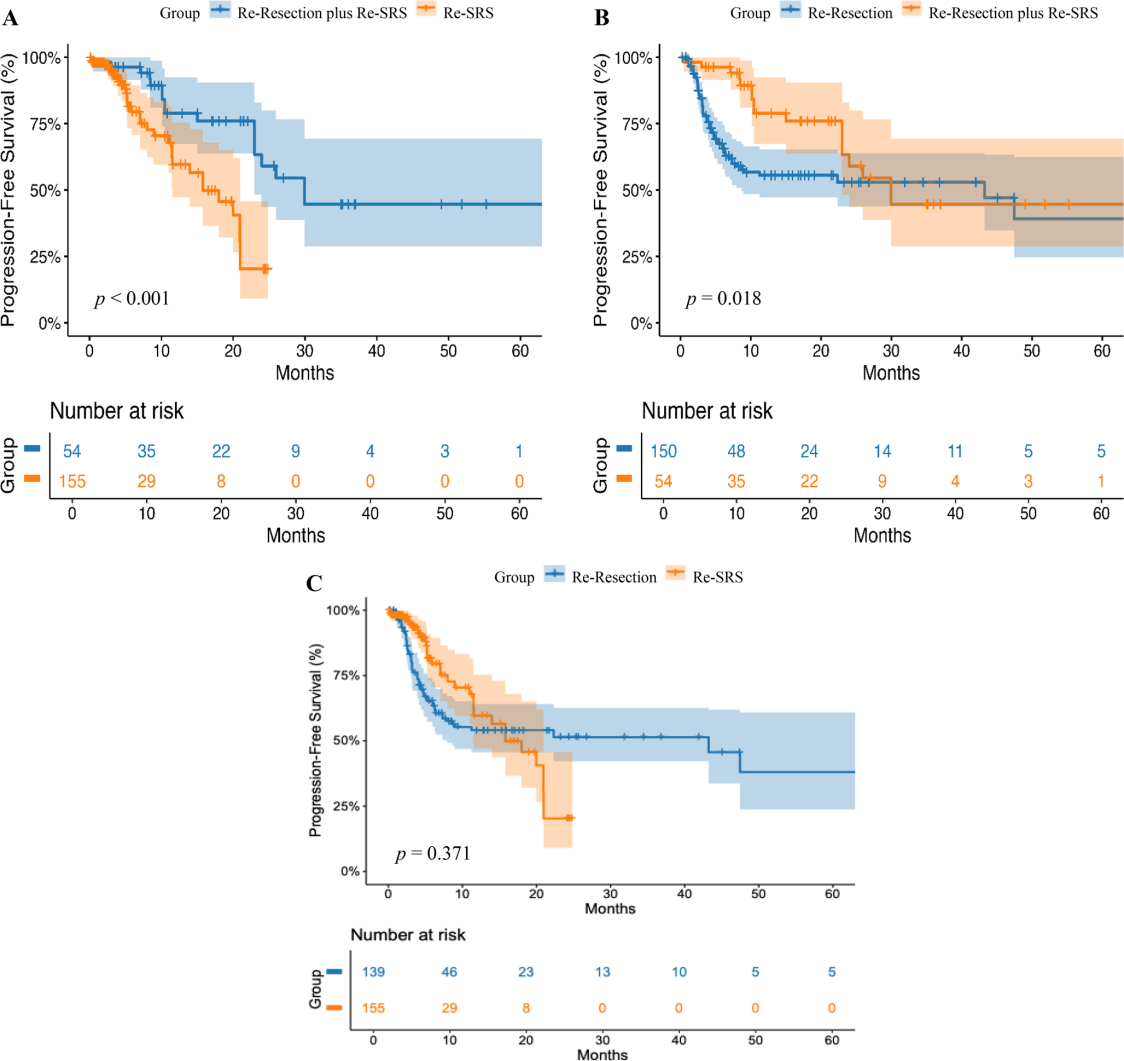
Kaplan-Meier analyses of PFS in patients with recurrent brain metastases according to treatment modality. (**A**) Kaplan-Meier curves comparing patients treated with local re-resection plus adjuvant re-SRS versus only re-SRS after recurrence. Patients who received combined local re-resection plus adjuvant re-SRS had significantly longer PFS compared to those treated with only SRS (43.23 months vs. 15.79 months; *p* < 0.001). (**B**) Kaplan-Meier curves comparing patients treated with only local re-resection versus local re-resection plus adjuvant SRS after recurrence. The group receiving local re-resection plus adjuvant re-SRS demonstrated significantly longer PFS (43.23 vs. 29.92 months; *p* = 0.018). (**C**) Kaplan-Meier curves comparing patients treated with only local re-resection versus only re-SRS after recurrence. No statistically significant difference between these two groups in PFS was observed (43.23 months versus 15.79; *p* = 0.371). Shaded areas indicate 95% confidence intervals (CIs). The number of patients at risk at specified time points is shown below each plot. Tumor origin is not shown on the curves because origin-specific plots were unavailable for most cohorts

For PFS after local recurrence, IPD were available for 359 patients. The median PFS among patients who underwent local re-resection was 43.23 months (95% CI: 12.26–74.20), compared to 29.92 months (95% CI: 21.84–38.00) in those who received re-resection followed by adjuvant SRS. Patients who underwent re-resection alone had a statistically significant longer PFS, than those who received postoperative SRS (HR = 0.529; 95% CI: 0.307–0.908; *p* = 0.018). The median PFS in patients treated with re-resection plus SRS was 29.92 months (95% CI: 21.84–38.00), compared to 15.79 months (95% CI: 9.96–21.61) in those who received SRS alone after recurrence. This difference was statistically significant (HR: 3.031; 95% CI: 1.58–5.83, *p* < 0.001). In contrast, there was no statistically significant difference in PFS after recurrence between patients treated with re-resection alone (43.23; 95% CI: 12.22–74.24), and those treated with SRS alone (15.79 months; 95% CI: 9.96–21.61) (HR = 0.821; 95% CI: 0.53–1.26; *p* = 0.371). Kaplan-Meier curves for PFS after recurrence are shown in Fig. 4.

### Bias and quality evaluation

**Fig. 5:**
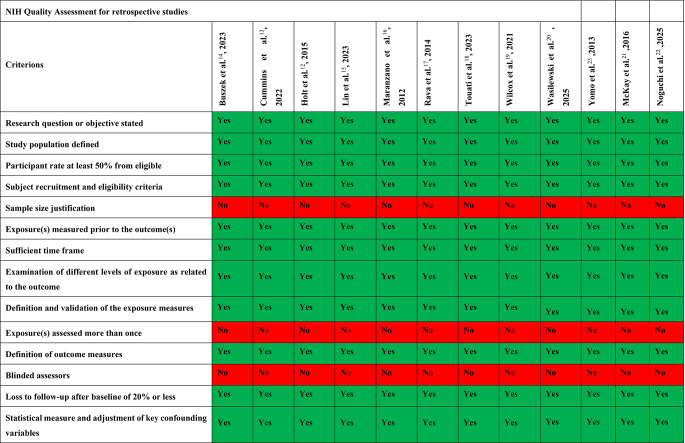
The figure presents the NIH-QAT assessment for the twelve studies included in this meta-analysis. It evaluates the methodological rigor of each study based on the NIH quality assessment criteria. Each study was assessed according to a set of predefined questions. Responses marked as “Yes” indicate that the respective criterion was met, while those marked as “No” signify that the criterion was not fulfilled

The NIH-QAT for retrospective studies was applied to nine publications: Buszek et al. [14], Cummins et al. [13], Holt et al. [12], Lin et al. [15], Maranzano et al. [16], Rava et al. [17], Touati et al. [18], Wilcox et al. [19], Wasilewski et al. [20], MyKac et al. [21], Noguchi et al. [22], and Yomo et al. [23]. Across these studies, objectives were clearly stated, populations well described, and ≥50% of eligible patients were enrolled. Recruitment and inclusion criteria were presented in a transparent manner, exposures were assessed prior to the endpoints, and the observation periods were sufficiently extensive to validly reflect therapeutic courses and clinical endpoints. Furthermore, all studies incorporated varying exposure levels into their evaluation, defined and validated exposure measurements in a comprehensible manner, and clearly defined the endpoints. The proportion of patients with incomplete follow-up also remained below 20% in all cases, and potential confounders were addressed statistically (Fig. 5).

## Discussion

This study analyzed treatment strategies for locally recurrent BM, including re-resection, re-SRS, and systemic therapies, and their effect on OS and PFS after recurrence. Using pooled meta-analysis and IPD from 776 patients, local re-resection improved median OS over no surgery (14.74 vs. 10.34 months; HR: 0.664; *p* < 0.001) and re-SRS alone (14.74 vs. 10.97 months; HR: 0.62; *p* < 0.001). Among 359 patients, local re-resection alone showed longer PFS than re-resection with adjuvant SRS (43.23 vs. 29.92 months; HR: 0.529; *p* = 0.018). Re-resection plus SRS had longer PFS than SRS alone (29.92 vs. 15.79 months; HR: 3.031; *p* < 0.001). No significant PFS difference was seen between re-resection alone and SRS alone (*p* = 0.371). Salvage treatment at recurrence is uncommon, as only a minority of patients meet strict candidacy criteria for either local re-resection or re-SRS. This clinical rarity limits the size and uniformity of available studies, which is why the literature covers diverse primary tumor origins and prior treatments [26–28]. We therefore interpreted results as comparative effectiveness across studies with heterogenous reporting.

These findings are consistent with prior series reporting OS benefits with salvage re-resection [13]. Selection factors such as lesion location and size, primary diagnosis, and systemic disease status influence whether patients receive re-resection, re-SRS, or re-resection with adjuvant re-SRS. However, these variables were not uniformly available or could not be linked to post-recurrence survival data by treatment across studies. Re-resection is most attractive for patients with a single dominant, surgically accessible lesion, mass effect, and adequate KPS, particularly when GTR is feasible. Re-SRS is preferred when surgical morbidity is high, lesion burden is limited, or prior surgery after adequate interval since prior radiation [15, 20, 27, 29–31]. 

 [13, 15, 19, 20, 27, 29–31] Patients who achieved GTR after local recurrence had significantly longer OS compared to those with STR or incomplete re-resection. In 213 patients, Lin et al. [15] defined GTR by a residual tumor volume cut-off of 0.12 cm^3^ and found improved OS versus larger residual volumes or incomplete re-resection [15]. Our results support these findings and emphasize the importance of achieving maximal cytoreduction to improve OS. Local re-resection with adjuvant SRS yielded longer PFS than SRS alone [17]. This aligns with Touati et al. [18], who observed improved PFS in patients treated with local re-resection followed by adjuvant SRS compared with those without postoperative radiotherapy. Adjuvant SRS after re-resection may improve PFS in selected patients and can delay progression, whereas re-resection immediately relieves mass effect and improves function. Surveillance schedules and criteria varied, and subsequent systemic therapies may extend OS independent of intracranial PFS [18, 19]. Given non harmonized covariate reporting, we report both endpoints but do not treat PFS as a surrogate for OS.

This study highlights local re-resection as an important component of the treatment strategy for recurrent BM. Beyond OS, surgery relieves mass effect and symptoms and can improve neurological function and quality of life (QoL), especially in large space occupying lesions or acute deterioration [32–34]. Although OS was longer with local re-resection, this finding should be interpreted in the context of patient selection, EoR and the tailored use of adjuvant therapies, including radiotherapy. We did not observe an OS advantage from postoperative reirradiation, consistent with ongoing controversy [13, 15, 18, 19]. This may reflect differences in tumor biology, primary tumor type, KPS, and selection of radiation-resistant clones after prior radiotherapy [35–38]. Our findings underscore the importance of a multidisciplinary and individualized approach to the management of recurrent BM, integrating neurosurgical, oncological, and radiotherapeutic expertise [19, 31, 39].

There are also several limitations to our study. To probe robustness to between study heterogeneity, we performed sensitivity checks that excluded disease restricted cohorts and cohorts that did not separate outcomes by salvage treatment modality, and we also restricted the pool to studies reporting re-resection and re-SRS only. Across these restrictions, the direction of the primary contrasts was unchanged, although precision decreased as expected. A central limitation is that lesion location, lesion size, primary diagnosis, and systemic disease status were not consistently reported or were not linkable to post recurrence survival data across studies. Consequently, adjusted or subgroup-specific pooled estimates could not be calculated, and residual confounding by indication and surveillance bias for PFS or OS cannot be excluded. The heterogeneity across the included studies and patient populations including differences in patient characteristics, primary tumor types, genetic mutation profiles of brain metastases, center-specific expertise in managing recurrent brain metastases, varying radiotherapy dosing protocols may limit the generalizability of our findings. Additionally, data on treatment at initial diagnosis (e.g., primary stereotactic radiotherapy only, surgical resection and adjuvant stereotactic radiotherapy) were not uniformly available. The limited number of patients in specific subgroups, particularly those receiving adjuvant systemic therapy, further reduces the interpretability of subgroup analyses. Moreover, both pre- and postoperative KPS are critical determinants in treatment allocation. Patients with higher KPS are more likely to undergo surgical intervention, potentially introducing outcome bias in favor of surgery. Re-resection might be associated with increased complications and the present pooled dataset represents a carefully selected cohort. Hence, this pooled cohort might be limited by selection bias and further large-scale data investigating safety profile for re-resections are needed [27]. Safety reporting was incomplete in most included studies, precluding pooled estimates. Procedure-related morbidity after re-resection and symptomatic radionecrosis after reirradiation are central to decision making. Future studies should prospectively capture complications, steroid dependence and symptomatic radionecrosis to enable balanced risk-benefit assessments [19, 31, 39]. Another limitation is incomplete and non-linkable reporting of tumor origin in many included studies. As a result, origin-adjusted or origin-specific pooled estimates could not be calculated and origin bias may persist if the mix of primary tumors differed between salvage treatment groups or if tumor biology modified treatment effects. These factors limit generalizability to any single origin and underscore the need for future datasets with origin-linked time-to-event reporting. Taken together, future prospective studies are needed to evaluate salvage strategies in recurrent BM and should prespecify GPA strata, molecular profiles, radiotherapy dose and fractionation, and EoR categories, use protocolized imaging schedules with consensus progression criteria, and collect standardized endpoints including patient-reported outcomes.

## Conclusion

In recurrent BM, local re-resection was associated with longer OS than no surgery and re-SRS, with the most favorable outcomes when GTR could be achieved. We observed longer PFS with re-resection plus adjuvant re-SRS than with re-SRS alone, whereas re-resection alone yielded longer PFS than re-resection plus adjuvant re-SRS. This pattern likely reflects differences in case selection and closer postoperative imaging rather than a uniform biological effect. We did not find a clear OS advantage attributable to postoperative re-SRS. These findings support individualized management that takes into account tumor resectability, EoR, of mass effect, KPS, prior radiation exposure and patient preferences within a multidisciplinary team.

## Data Availability

No datasets were generated or analysed during the current study.
